# Wave separation analysis-derived indexes obtained from radial and carotid tonometry in healthy pregnancy and pregnancy-associated hypertension: Comparison with pulse wave analysis-derived indexes

**DOI:** 10.3389/fcvm.2022.997452

**Published:** 2022-11-01

**Authors:** María M. Pereira, Juan Torrado, Joshua Bock, Claudio Sosa, Alejandro Diaz, Daniel Bia, Yanina Zócalo

**Affiliations:** ^1^Department of Obstetrics and Gynecology, BronxCare Hospital Center a Clinical Affiliate of Mt Sinai Health Systems and Academic Affiliate of Icahn School of Medicine, Bronx, NY, United States; ^2^Department of Internal Medicine, Jacobi Medical Center, Albert Einstein College of Medicine, Bronx, NY, United States; ^3^Department of Internal Medicine, Montefiore Medical Center, Albert Einstein College of Medicine, Bronx, NY, United States; ^4^Department of Obstetrics and Gynecology “C”, Pereira-Rossell Hospital, School of Medicine, Republic University, Montevideo, Uruguay; ^5^Consejo Nacional de Investigaciones Científicas y Técnicas, Tandil, Argentina; ^6^Centro Universitario de Investigación, Innovación y Diagnóstico Arterial (CUiiDARTE), Department of Physiology, School of Medicine, Republic University, Montevideo, Uruguay

**Keywords:** applanation tonometry, gestational hypertension, pregnancy, preeclampsia, pulse wave analysis, wave separation analysis

## Abstract

**Background:**

Increased wave reflections assessed by pulse wave analysis (PWA) was proposed as one of the potential culprits of hypertension seen in women with pregnancy-associated hypertension (PAH). However, this statement has never been confirmed with “Wave Separation Analysis” (WSA), a more sophisticated mathematical approach that analyzes the amplitude and interaction between forward and backward aortic pressure waveform components.

**Objective:**

To characterize potential changes in pressure wave components of PAH compared to healthy non-pregnant (NP) women and women with normal pregnancies (HP) by using WSA and compared these findings with PWA-derived indexes; secondarily, to evaluate differences in WSA-derived indexes between subgroups of PAH (i.e., preeclampsia [PE] and gestational hypertension [GH]).

**Methods:**

Using radial and carotid applanation tonometry, we quantified in HP (*n* = 10), PAH (*n* = 16), and NP (*n* = 401): (i) PWA-derived indexes; (ii) WSA-derived indexes: forward (Pf) and backward (Pb) waveform components, backward component arrival time (PbAT), reflection magnitude (RM = Pb/Pf) and index [RIx = Pb/(Pf + Pb)].

**Results:**

While PAH was associated with a higher Pf compared to HP and NP, Pb and PbAT were similar between the groups. Both GH and PE showed a higher Pf compared to HP, but only PE had a trend of presenting with higher Pb and lower PbAT compared to the other groups. Finally, PAH showed a trend of having lower RM and RIx compared to NP and HP, with no differences between GH and PE.

**Conclusion:**

PAH was associated with higher Pf, but not higher Pb, compared to NP and HP, although PE also demonstrated a trend of higher Pb.

## Introduction

Healthy pregnancy (HP) is characterized by a myriad of changes in the structure and function of the maternal cardiovascular system that are evident early during pregnancy (1, 2). These modifications, including an enhancement of endothelial function, a drop in the peripheral vascular resistance and aortic stiffness, and the preservation of the stiffness gradient (3) are all critical to ensure a sufficient utero-placental perfusion (to meet the fetal metabolic demands) with no increments in the blood pressure (2–4).

Recent evidence supports the “cardiovascular maladaptation hypothesis” of preeclampsia (PE]), in which the inability of the great arteries to adapt properly (arterial impairment) imposes additional hemodynamic loads on the maternal and fetal circulations leading to further hemodynamic derangements (3, 5). In this context, the possibility of measuring the level or degree of cardiovascular maladaptation to pregnancy that can occur in different pregnancy-associated hypertension (PAH) disorders not only would allow for a comprehensive understanding of their pathophysiology but can also be an opportunity to generate new preventive diagnostic tools and treatments (6–8). Among different tools, the examination of central pressure waveform-derived indexes, using “pressure-only approaches for waveform analysis,” has been promising (9–15).

**FIGURE 1:**
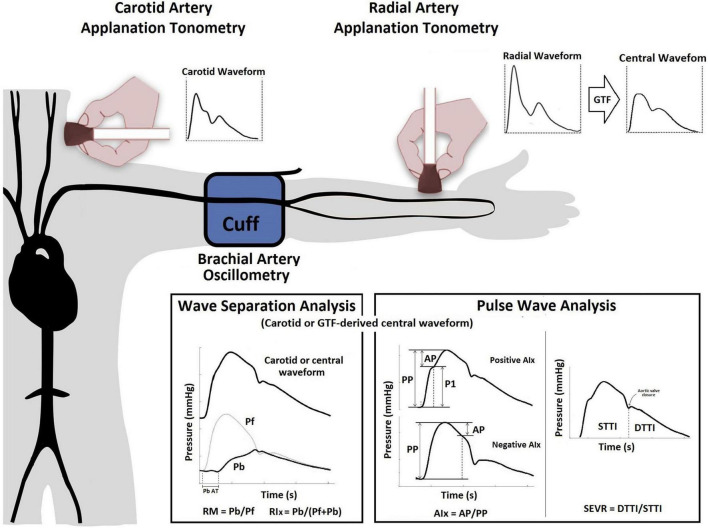
Use of applanation tonometry (radial or carotid) for the assessment of PWA and WSA. PWA: The AP represents the direct augmentation level caused by wave reflections (a positive AP indicates “additional” pressure arising from wave reflections) (36), and is calculated from the inflection point in the pressure waveform (systolic phase) that “points out or identifies” the arrival of the reflected component to the aortic root (37). AIx (either a positive or negative value) is calculated as AP/aoPP and is considered a surrogate index of wave reflection, although it also depends on factors like HR, vascular tone, or LV function (22). SEVR, quantified as the ratio between aortic diastolic and systolic tension-time areas or indexes [STTI and DTTI], represents a parameter of myocardial oxygen supply and demand that can be altered by changes in diastolic and systolic pressures as a result of changes in the arrival of the backward pulse wave (16). WSA: The Pf represents the integration of the forward wave arising from the ventricle and re-reflections of backward propagating waves at the ventricular-aorta interface. Using Pf and Pb, the reflection magnitude (RM = Pb/Pf) and index [RIx = (Pb/(Pf + Pb)] can be obtained (13, 38). Abbreviations as in text.

The most widely used model to analyze the central pressure waveform is based on the “Pulse Wave Analysis” (PWA) approach, which allows for the quantification of augmentation pressure (AP) and augmentation index (AIx), along with their heart rate (HR) corrected versions (APHR75, AIxHR75, respectively), and subendocardial viability ratio (SEVR) (15, 16) (Figure 1). The theoretical concept underlying PWA is that forward waves generated by the left ventricle (LV) travel to the periphery, reflect distally, and travel back fast enough to collide with forward pressure (Pf) waves, thereby augmenting the (central) aortic systolic and pulse pressures (aoSBP, aoPP, respectively). Several groups quantified PWA-derived indexes in pregnant women in order to characterize potential specific pregnancy-induced physiological variations and differences between HP and PAH states, including PE (6, 8, 17–19). While some investigators found that PAH states (in particular PE) are associated with a higher AIx (or AIxHR75) in comparison to HP (17, 18), others found no differences in these indexes among these pregnant women (8, 20, 21). Several biological and/or methodological reasons may explain this controversy.

More recently, other “pressure-only approaches” for waveform analysis, such as “Wave Separation Analysis” (WSA), have been introduced. In this approach, the central pressure waveform, which integrates different forward and backward propagating waves, is decomposed into single forward (Pf) and backward (Pb) components (Figure 1). Several clinical studies have shown that WSA could provide valuable clinical information about hemodynamics and cardiovascular efficiency (13, 15, 22). However, to the best of our knowledge, it remains unknown whether WSA-derived indexes change in pregnancy, particularly, in women with PAH. As PWA and WSA-derived indexes are influenced by common variables (e.g., arterial stiffness), the variables derived by these approaches could show association between one another. Nevertheless, the information provided by WSA-derived indexes could differ, complement, or even offer better information during normal pregnancy or pregnancy complicated by PAH than PWA-derived indexes. Besides, the WSA-derived indexes could be useful for the identification of variations associated with PAH states. Finally, it is noteworthy that several techniques (e.g., applanation tonometry, plethysmography) and mathematical methods [e.g., direct carotid or distal-arteries recordings associated to a general transfer function (GTF)] have been proposed to perform PWA and WSA (22, 23). Our group has previously worked in this area, demonstrating that values of a single WSA- or PWA-derived index (e.g., AIx, Pf) could markedly differ based on the technique and recording site (e.g., carotid [CT] vs. radial [RT] applanation tonometry) (23, 24).

In this context, we sought to analyze (1) the levels of association between WSA and PWA derived indexes and (2) to characterize and compare WSA-derived indexes in HP and PAH. Additionally, we identified potential differences between subgroups of PAH patients (i.e., PE and GH). Considering the different approaches to obtaining these non-invasive indexes, we employed the gold-standard technique (i.e., applanation tonometry) with two different ways of recording: RT (indirect method that requires a GTF) and CT (direct method that does not require a GTF).

## Materials and methods

### Subjects

This was a cross-sectional study involving NP, and pregnant women from our CUiiDARTE Project database (25–28). Cardiovascular evaluation in the CUiiDARTE Project involves a stepwise protocol using several equipment and devices that measure structural and functional properties of central and peripheral arteries, as well as hemodynamic variables (25–28). All procedures were conducted in agreement with the Declaration of Helsinki and approved by the Institution’s Ethics Committee. Written informed consent was obtained prior to the examination. The data presented in this work was obtained in a study protocol, which has already given rise to a recent publication related to other aspects of arterial behavior in normal and pathophysiological circumstances (7). Healthy NP (*n* = 401) women were selected to be matched for age and global cardiovascular risk factors (CRFs) with the below-mentioned pregnant women. By using propensity score matching methods, an efficient matching and balance is created among the mentioned covariates, thereby, minimizing or entirely removing their confounding effect (29). HP women (*n* = 10), without known family history of premature cardiovascular disease (CVD), were recruited from the routine antenatal clinic. All women had uncomplicated pregnancies before and during the study. Women with PAH (*n* = 16) were recruited from the antenatal hospital ward, where they were admitted due to mild hypertension (brachial artery blood pressure [baBP] 140/90 to 149/109 mmHg).

According to the Bulletin of the American College of Obstetricians and Gynecologists (30), PAH is defined as baSBP of 140 mm Hg or more or baDBP of 90 mm Hg or more on two occasions at least 4 h apart after 20 weeks of gestation in a woman with a previously normal BP. Depending on whether there was significant proteinuria (≥300 mg per 24-h urine collection), patients were further classified as PE or GH, respectively, since all patients included in the study had no evidence of severe features. Laboratory samples were obtained prior to the study enrollment. A clinical interview, together with the anthropometric evaluation (body weight [BW], height [BH] and mass index [BMI]) enabled us to assess for CRF exposure (i.e., family history of CVD, obesity, dyslipidemia).

### Non-invasive arterial evaluation: Central blood pressure levels and wave separation analysis- and pulse wave analysis-derived indexes

Central aortic blood pressure (aoBP) levels and waveforms were obtained (random order) using a commercially available device: SphygmoCor-CvMS (v.9, AtCor-Medical, Sydney, NSW, Australia). Subjects were instructed to lie in a left lateral position (to avoid vena cava compression by the uterus) in a temperature-controlled (21–23°C) room, for at least 15 min, in order to establish stable hemodynamic conditions. The aoBP waveforms were derived from, (i) radial artery (applying a GTF) and (ii) carotid artery (directly) manual tonometric recordings (Figure 1). Carotid pulse waveforms were assumed to be identical to the aortic ones (due to the proximity of the arterial sites) (31). Thus, a GTF was not applied to obtain central waveforms from carotid recordings. Only accurate waveforms on visual inspection and high-quality recordings (in-device quality control [operator] index > 95%) were considered.

The SphygmoCor device was used for WSA and PWA. A detailed explanation of the method used for waveform analysis based on recorded carotid waveforms and mathematically-derived aortic waveforms was included as Supplementary material in a previous study (24). As was previously published, the absolute and relative intra- (repeatability) and interobserver (reproducibility) variability of aoBP levels and waveform-derived indexes was analyzed considering different methodological approaches (RT and CT) (23, 24). In all cases, relative inter- and intraobserver variability was < 6%.

Using applanation tonometry and two recording sites (carotid and radial), we quantified: (i) PWA-derived indexes: AP, APHR75, AIx, AIxHR75, STTI, DTTI, SEVR, and (ii) WSA-derived indexes: Pf, Pb, Pb AT (backward pressure arrival time to central pressure waveform), RM, and RIx (Figure 1 and Supplementary Table 1). Recorded waveforms were calibrated using brachial artery diastolic (baDBP) and mean blood pressures [baMBP = baDBP + (baSBP-baDBP)/3], where baSBP is brachial artery systolic blood pressure (23, 24).

### Data analysis

First, after descriptive statistics were computed (Table 1 and Supplementary Table 2), aimed at determining whether the WSA-derived indexes are related to PWA-derived indexes, we analyzed the level of association between them by assessing simple bivariate correlation (Pearson coefficients, *r*) (Table 2). Second, one-way analysis of covariance (ANCOVA) with multiple adjusted comparisons was used for the evaluation of differences in cardiovascular variables. Demographic characteristics (age), anthropometric measurements (BW, BH, BMI), CRFs exposure and medication use were categorized as adjustment variables. Considering the relatively small sample sizes of the HP and PAH groups, we performed bootstrapping of the samples (both, for correlations and ANCOVA) as a strategy to evaluate whether potential statistical differences observed between the study groups are maintained even after analyzing different random sampling settings. To this end, bootstrap-derived 95% confidence intervals (1,000 samples) were obtained applying bias-corrected and accelerated methods for computing confidence interval limits (32). In other words, with this statistical mechanism, any initial *p* < 0.05 may no longer be significant after the “fictional random re-sampling” (i.e., bootstrapping). This type of test is a conservative approach that obligates the investigators to consider only those significant *p*-values that replicate in both statistical scenarios (i.e., the actual sample and bootstrapping sampling). As secondary analysis, we further investigate differences between four groups (NP, HP, GH, PE) by discriminating between women with PE and GH within the PAH group.

**TABLE 1:** Clinical and blood pressure levels and waveform-derived indexes according to the study groups.

	Non-pregnant women				Healthy pregnant women				Pregnancy-associated hypertension			
		
	MV	*SE*	Min.	Max.	MV	*SE*	Min.	Max.	MV	*SE*	Min.	Max.
Age [years]	22.84	0.30	18	41	29.40	1.95	21.00	40	32.25	1.56	20.00	40.00
BMI [Kg/m^2^]	23.04	0.22	16.50	48.77	27.13	1.13	20.72	31.23	34.16	1.78	21.77	48.27
Hypertension [%]	4.0%				0.0%				100.0%			
BP treatment (%)	1.7%				0.0%				12.5%			
Dyslipidemia (%)	7.2%				20.0%				0.0%			
**Basic hemodynamics**												
baSBP [mmHg]	118	1	85	177	114	2	106	121	127	3	98	143
baDBP [mmHg]	69		49	103	65	3	52	85	75	2	59	93
HR [bpm]	69	1	43	102	77	8	51	93	82	3	69	95
**Radial applanation tonometry**												
aoSBP [mmHg]	103	1	79	156	99	2	90	110	112	3	84	126
aoDBP [mmHg]	70		47	103	67	3	55	87	78	2	60	94
aoPP [mmHg]	33		17	68	33	2	22	46	33	2	24	44
AP [mmHg]	2		–13	18	3	1	–2	6	4	1	–3	13
AIx	7	1	–26	41	9	3	–6	18	12	3	–11	31
SEVR	144	1	77	261	128	11	86	183	123	7	87	177
Pf [mmHg]	30		15	68	28	3	16	42	29	1	22	39
Pb [mmHg]	14		5	39	14	1	8	22	13	1	9	20
Pb AT [ms]	252	1	180	477	240	8	203	281	244	5	211	281
RM	0.48	0.01	0.17	1.00	0.53	0.04	0.38	0.77	0.45	0.02	0.31	0.65
RIx	0.32	0.00	0.14	0.50	0.34	0.02	0.27	0.44	0.31	0.01	0.24	0.39
**Carotid applanation tonometry**												
aoSBP [mmHg]	110	1	73	164	112	7	96	146	117	5	81	147
aoDBP [mmHg]	66	1	48	100	69	5	53	85	72	3	54	95
aoPP [mmHg]	44	1	10	80	43	9	21	84	45	4	27	85
AP [mmHg]	–6	1	–32	32	–8	6	–33	4	–4	4	–50	14
AIx	–12	1	–47	40	–12	9	–39	10	–5	7	–58	34
SEVR	145	2	93	223	130	19	70	187	121	4	86	151
Pf [mmHg]	44	1	8	107	34	5	19	46	45	5	24	85
Pb [mmHg]	16		6	31	16	3	11	26	17	2	9	28
Pb AT [ms]	270	3	211	445	262	14	219	289	258	13	211	375
RM	0.41	0.01	0.15	0.96	0.50	0.07	0.24	0.63	0.43	0.05	0.15	0.70
RIx	0.28	0.00	0.13	0.49	0.32	0.04	0.19	0.39	0.29	0.03	0.13	0.41

MV, mean value; SE, standard error; Min, minimal value; Max, maximal value; BMI, body mass index; SBP, PP, DBP, MBP, systolic pulse, diastolic and mean blood pressure; ao, aortic; ba, brachial artery; ra, radial artery; BP, blood pressure; Pf, central forward pulse pressure height; Pb, central backward pulse pressure height; PbAT, central backward arrival time; AIx, augmentation index; AP, central augmented pressure; SEVR, sub-endocardial viability ratio; HR, heart rate; RM, reflection magnitude; RIx, reflection index. Sample size: non-pregnant women (*n* = 401), healthy pregnant (*n* = 10), pregnancy-associated hypertension (*n* = 16).

**TABLE 2:** Association between hemodynamic and waveform derived indexes.

		Radial applanation tonometry					Carotid applanation tonometry				
			
		Pf (mmHg)	Pb (mmHg)	Pb AT (ms)	RM	RIx	Pf (mmHg)	Pb (mmHg)	Pb AT (ms)	RM	RIx
**Radial applanation tonometry**											
baSBP (mmHg)	*r*	0.786	0.545	0.046	–0.353	–0.367	0.507	0.291	0.075	–0.261	–0.252
	*p*	<0.001	<0.001	0.193	<0.001	<0.001	<0.001	<0.001	0.172	<0.001	0.001
baDBP (mmHg)	*r*	–0.439	–0.126	–0.304	0.345	0.367	–0.276	–0.066	–0.361	0.257	0.256
	*p*	<0.001	0.009	<0.001	<0.001	<0.001	<0.001	0.201	<0.001	<0.001	<0.001
baMBP (mmHg)	*r*	–0.156	0.129	–0.284	0.266	0.282	–0.020	0.123	–0.327	0.164	0.160
	*p*	0.002	0.008	<0.001	<0.001	<0.001	0.401	0.059	<0.001	0.019	0.021
aoSBP (mmHg)	*r*	0.372	0.536	–0.088	0.101	0.093	0.343	0.352	–0.099	–0.010	–0.014
	*p*	<0.001	<0.001	0.048	0.028	0.041	<0.001	<0.001	0.107	0.450	0.428
aoDBP (mmHg)	*r*	–0.439	–0.135	–0.321	0.334	0.356	–0.261	–0.079	–0.360	0.231	0.229
	*p*	<0.001	0.005	<0.001	<0.001	<0.001	<0.001	0.160	<0.001	0.002	0.002
AP (mmHg)	*r*	–0.504	0.282	–0.195	0.820	0.819	–0.341	0.112	–0.253	0.476	0.443
	*p*	<0.001	<0.001	<0.001	<0.001	<0.001	<0.001	0.078	0.001	<0.001	<0.001
AIx (%)	*r*	–0.570	0.194	–0.168	0.858	0.861	–0.341	0.109	–0.210	0.501	0.467
	*p*	<0.001	<0.001	0.001	<0.001	<0.001	<0.001	0.084	0.004	<0.001	<0.001
SEVR	*r*	0.012	0.279	0.203	0.290	0.297	0.078	0.346	0.161	0.251	0.261
	*p*	0.407	<0.001	<0.001	<0.001	<0.001	0.161	<0.001	0.020	0.001	<0.001
Pf(mmHg)	*r*	1	0.546	0.296	–0.577	–0.602	0.554	0.323	0.221	–0.316	–0.301
	*p*	——-	<0.001	<0.001	<0.001	<0.001	<0.001	<0.001	0.003	<0.001	<0.001
Pb (mmHg)	*r*	0.546	1	0.024	0.320	0.307	0.318	0.473	0.120	0.127	0.105
	*p*	<0.001	——-	0.329	<0.001	<0.001	<0.001	<0.001	0.066	0.056	0.094
Pb AT (ms)	*r*	0.296	0.024	1	–0.239	–0.264	0.030	0.183	0.300	0.076	0.093
	*p*	<0.001	0.329	——-	<0.001	<0.001	0.353	0.011	<0.001	0.171	0.121
RM	*r*	–0.577	0.320	–0.239	1	0.991	–0.358	0.072	–0.128	0.527	0.481
	*p*	<0.001	<0.001	<0.001	——-	<0.001	<0.001	0.183	0.054	<0.001	<0.001
RIx	*r*	–0.602	0.307	–0.264	0.991	1	–0.358	0.066	–0.121	0.513	0.473
	*p*	<0.001	<0.001	<0.001	<0.001	——-	<0.001	0.206	0.064	<0.001	<0.001
**Carotid applanation tonometry**											
baSBP (mmHg)	*r*	–0.068	0.002	–0.032	0.090	0.104	0.293	0.366	–0.006	0.019	0.020
	*p*	0.222	0.489	0.362	0.155	0.122	<0.001	<0.001	0.475	0.416	0.412
baDBP (mmHg)	*r*	–0.218	0.065	–0.157	0.294	0.300	–0.434	–0.187	–0.362	0.312	0.315
	*p*	0.007	0.233	0.038	<0.001	<0.001	<0.001	0.019	<0.001	<0.001	<0.001
baMBP (mmHg)	*r*	0.101	0.086	0.001	–0.047	–0.060	–0.263	–0.297	–0.050	0.010	0.010
	*p*	0.128	0.167	0.496	0.301	0.251	0.002	<0.001	0.290	0.458	0.456
aoSBP (mmHg)	*r*	0.425	0.443	0.018	–0.053	–0.070	0.653	0.556	0.050	–0.164	–0.174
	*p*	<0.001	<0.001	0.407	0.244	0.182	<0.001	<0.001	0.262	0.018	0.013
aoDBP (mmHg)	*r*	–0.283	–0.007	–0.177	0.307	0.307	–0.410	–0.189	–0.334	0.325	0.320
	*p*	<0.001	0.464	0.010	<0.001	<0.001	<0.001	0.008	<0.001	<0.001	<0.001
AP (mmHg)	*r*	–0.496	0.079	–0.078	0.601	0.607	–0.750	–0.052	–0.291	0.736	0.726
	*p*	0.000	0.152	0.156	<0.001	<0.001	<0.001	0.253	<0.001	<0.001	<0.001
AIx (%)	*r*	–0.444	0.152	–0.044	0.652	0.658	–0.633	0.112	–0.261	0.827	0.804
	*p*	<0.001	0.023	0.284	<0.001	<0.001	<0.001	0.078	<0.001	<0.001	<0.001
SEVR	*r*	0.184	0.283	0.319	0.129	0.134	–0.038	0.206	0.183	0.241	0.255
	*p*	0.008	<0.001	<0.001	0.046	0.040	0.314	0.004	0.010	0.001	0.001
Pf (mmHg)	*r*	0.554	0.318	0.030	–0.358	–0.358	1	0.424	0.453	–0.628	–0.651
	*p*	<0.001	<0.001	0.353	<0.001	<0.001	——-	<0.001	<0.001	<0.001	<0.001
Pb (mmHg)	*r*	0.323	0.473	0.183	0.072	0.066	0.424	1	–0.041	0.352	0.358
	*p*	<0.001	<0.001	0.011	0.183	0.206	<0.001	——-	0.300	<0.001	<0.001
Pb AT (ms)	*r*	0.221	0.120	0.300	–0.128	–0.121	0.453	–0.041	1	–0.405	–0.438
	*p*	0.003	0.066	<0.001	0.054	0.064	<0.001	0.300	——-	<0.001	<0.001
RM	*r*	–0.316	0.127	0.076	0.527	0.513	–0.628	0.352	–0.405	1	0.990
	*p*	<0.001	0.056	0.171	<0.001	<0.001	<0.001	<0.001	<0.001	——-	<0.001
RIx	*r*	–0.301	0.105	0.093	0.481	0.473	–0.651	0.358	–0.438	0.990	1
	*p*	<0.001	0.094	0.121	<0.001	<0.001	<0.001	<0.001	<0.001	<0.001	——-

r, Pearson coefficient; SBP, PP, DBP, MBP, systolic, pulse, diastolic and mean blood pressure; ao, aortic; ba, brachial artery; Pf, central forward pulse pressure height; Pb, central backward pulse pressure height; PbAT, central backward arrival time; Aix, augmentation index; AP, central augmented pressure; SEVR, sub-endocardial viability ratio; RM, reflection magnitude; RIx, reflection index.

Normality of the distribution of the data was examined using the Shapiro–Wilk test and Q-Q plots, with *P* < 0.05 indicating significant statistical differences. The statistical analyses were performed using the Statistical Package for Social Sciences (version 26.0). Evans’s Empirical Classification (“correlation strength”) was used for *r* interpretation as follows: <0.20, very weak; 0.20–0.39, weak; 0.40–0.59, moderate; 0.60–0.79, strong; ≥0.80, very strong (33).

## Results

Descriptive characteristics and baseline cardiovascular parameters of the study groups are presented in Table 1 and Supplementary Table 1. The mean gestational age at examination of all the pregnant women was 35 ± 3 weeks. No women had carotid plaques, diabetes, or family history of premature CVD (data presented elsewhere) (7).

### Wave separation analysis-derived indexes: Association with blood pressure levels and pulse wave analysis-derived indexes

Wave separation analysis-derived indexes (i.e., Pf, Pb, PbAT, RM, RIx) obtained through RT and CT showed significant associations (*p* < 0.05) with respect to baBP, aoBP levels, and PWA-derived indexes. As expected, Pf and Pb were positively associated with baSBP and aoSBP, which indicates that the greater the forward and backward wave components the greater the aoSBP (Table 2).

In general terms, there were statistically significant associations between the WSA- and PWA-derived indexes when considering RT and CT recordings. It is important to note though, when the WSA-derived indexes were compared to themselves (RT vs. CT recordings), despite the fact that the values showed statistically significant levels of association, the strengths of association were generally “moderate” (Pf: *r* = 0.554, *p* < 0.001; Pb: *r* = 0.473, *p* < 0.001; PbAT: *r* = 0.3, *p* < 0.001; RM, *r* = 0.527, *p* < 0.001; RIx, *r* = 0.473, *p* < 0.001) (Table 2).

#### Forward pressure

The levels of Pf and Pb were positively associated, which likely reflects the interrelationship between these parameters. In other words, a greater Pf component will determine a higher chance of wave reflections (a greater Pb), while a greater Pb component will raise the incident component (a greater Pf) by favoring transmission of wave reflections from the periphery to the center.

Conversely, the analysis between Pf and RM or RIx (parameters that assess the relative contribution of Pb to the resultant pressure wave) showed a negative association. In other words, the greater the Pf the smaller (relative) the contribution of Pb to the pulse pressure amplitude. Moreover, the associations between Pf and PWA-derived indexes were invariably negative, both when analyzing the “net” (AP, APHR75) or “relative” (AIx, AIxHR75) contribution of reflected waves (Table 2).

Of note, regardless of statistical significance (*p* < 0.05), the strength of association (*r* coefficient) between Pf and PWA-derived indexes was practically < 0.6 in all cases, having values ranging from “very weak” (*r*: 0.0–0.2), “weak” (*r*: 0.2–0.4) to “moderate” (*r*: 0.4–0.6) (Table 2).

#### Backward pressure (backward component arrival time)

As expected, Pb was positively associated with RM and RIx. Even though Pb was positively associated with APHR75 and AIxHR75, the strength of association was “very weak” (*r* < 0.2), which indicates that these parameters describe or characterize different hemodynamic phenomena (Table 2).

In addition, as predicted, a greater PbAT (late wave reflections arrival) was associated with a lower aoSBP, AP and AIx, and with a greater SEVR, with a strength of association for all being either “very weak” or “weak” (Table 2).

#### Reflection magnitude and reflection index

The levels of RM and RIx were significantly and positively associated with the aoSBP levels and with the PWA-derived indexes (AP, APHR75, Aix, and AIxHR75), with strengths of association that were “strong” (*r*: 0.6–0.8) or even “very strong” (*r*: 0.8–1.0). As a result, RM and RIx were the WSA-derived indexes that showed the strongest associations with PWA-derived indexes (i.e., AP and AIx) (Table 2).

### Central aortic blood pressure (carotid and radial records) of non-pregnant, healthy pregnancy, and pregnancy-associated hypertension

Regardless of the recording method (CT or RT), aoSBP was elevated in women with PAH compared to NP and HP, confirming that the hemodynamic disturbances in this group of patients are not exclusively a peripheral phenomenon (i.e., brachial), but also a central (i.e., aortic) abnormality (Table 3). Women with PE showed a trend of presenting with higher aoSBP levels compared to women with GH; however, these differences were only statistically significant when these parameters were obtained by RT (Table 3). Consequently, a methodological factor (CT vs. RT) could also be playing a role in these observations, either amplifying or blunting potential existing differences in the aoSBP of the PAH subgroups.

**TABLE 3:** Carotid and radial applanation tonometry-derived central blood pressure levels and waveform related parameters: comparison after adjustments (ANCOVA: 3 and 4 groups).

Variables: 3 groups	After adjustment						Pairwise comparisons					
				
		MV	SE	LL	UL		NP vs. HP		NP vs. PAH		HP vs. PAH	
**Carotid applanation tonometry**									
**aoSBP [mmHg]**	NP	109.43	1.03	107.40	111.46	**MD**	–0.048		–9.760		–9.712	
	HP	109.48	5.68	98.28	120.68	**P**	0.497		0.013		0.085	
	PAH	119.19	4.15	111.00	127.38	**Boot. P**	0.497		0.028		0.105	

**Pf [mmHg]**	NP	42.63	1.14	40.38	44.88	**MD**	6.570		–11.005		–17.575	
	HP	36.06	6.58	23.07	49.05	**P**	0.164		0.009		0.013	
	PAH	53.63	4.40	44.94	62.32	**Boot. P**	0.148		0.019		0.015	

**Pb [mmHg]**	NP	16.39	0.37	15.65	17.13	**MD**	0.540		–1.745		–2.285	
	HP	15.85	2.15	11.60	20.10	**P**	0.403		0.125		0.188	
	PAH	18.13	1.44	15.29	20.98	**Boot. P**	0.441		0.160		0.233	

**Pb AT [ms]**	NP	269.26	2.67	264.00	274.52	**MD**	2.154		1.414		–0.740	
	HP	267.11	15.33	236.84	297.37	**P**	0.445		0.448		0.484	
	PAH	267.85	10.26	247.60	288.10	**Boot. P**	0.424		0.458		0.487	

**RM**	NP	0.413	0.010	0.394	0.433	**MD**	–0.049		0.048		0.096	
	HP	0.462	0.056	0.351	0.573	**P**	0.197		0.115		0.076	
	PAH	0.366	0.038	0.291	0.440	**Boot. P**	0.294		0.180		0.173	

**RIx**	NP	0.287	0.005	0.277	0.297	**MD**	–0.021		0.030		0.050	
	HP	0.308	0.028	0.252	0.363	**P**	0.239		0.066		0.069	
	PAH	0.257	0.019	0.220	0.294	**Boot. P**	0.322		0.149		0.148	

**Radial applanation tonometry**									
**aoSBP [mmHg]**	NP	102.810	0.532	101.763	103.856	**MD**	5.377		–8.673		–14.050	
	HP	97.433	3.160	91.218	103.648	**P**	0.047		0.001		0.000	
	PAH	111.483	2.603	106.364	116.602	**Boot. P**	0.020		0.002		0.000	

**Pf [mmHg]**	NP	29.906	0.447	29.027	30.785	**MD**	1.501		–4.222		–5.723	
	HP	28.405	2.633	23.227	33.583	**P**	0.287		0.029		0.048	
	PAH	34.128	2.167	29.866	38.389	**Boot. P**	0.296		0.006		0.029	

**Pb [mmHg]**	NP	13.797	0.185	13.433	14.161	**MD**	–0.185		0.434		0.619	
	HP	13.982	1.090	11.838	16.126	**P**	0.434		0.319		0.331	
	PAH	13.363	0.897	11.599	15.127	**Boot. P**	0.433		0.326		0.326	

**Pb AT [ms]**	NP	251.908	1.351	249.250	254.565	**MD**	9.470		7.020		–2.450	
	HP	242.437	7.958	226.786	258.089	**P**	0.121		0.148		0.407	
	PAH	244.888	6.550	232.006	257.770	**Boot. P**	0.129		0.084		0.400	

**RM**	NP	0.481	0.006	0.469	0.492	**MD**	–0.034		0.097		0.131	
	HP	0.515	0.035	0.447	0.583	**P**	0.332		0.001		0.004	
	PAH	0.384	0.029	0.327	0.440	**Boot. P**	0.485		0.002		0.016	

**RIx**	NP	0.321	0.003	0.315	0.326	**MD**	–0.015		0.041		0.056*	
	HP	0.335	0.015	0.305	0.366	**P**	0.171		0.001		0.003	
	PAH	0.279	0.013	0.254	0.304	**Boot. P**	0.214		<0.001		0.004	

**Variables:** **4 groups**		**After adjustment**					**Pairwise comparisons**					
				
		**MV**	**SE**	**LL**	**UL**		**NP vs. HP**	**NP vs. GH**	**NP vs. PE**	**HP vs. GH**	**HP vs. PE**	**GH vs. PE**

**Carotid applanation tonometry**												
**aoSBP [mmHg]**	NP	109.4	1.0	107.4	111.5	**MD**	–0.067	–7.365	–12.745	–7.298	–12.678	–5.380
	HP	109.5	5.7	98.3	120.7	**P**	0.495	0.091	0.018	0.177	0.062	0.241
	GE	116.8	5.4	106.2	127.4	**Boot. P**	0.497	0.169	0.004	0.233	0.052	0.269
	PE	122.2	5.9	110.5	133.9		—-	—-	—-	—-	—-	—-

**Pf [mmHg]**	NP	42.6	1.1	40.4	44.9	**MD**	6.624	–14.185	–7.020	–20.809	–13.644	7.166
	HP	36.0	6.6	23.0	49.0	**P**	0.162	0.008	0.138	0.009	0.066	0.187
	GH	56.8	5.7	45.6	68.0	**Boot. P**	0.139	0.019	0.143	0.011	0.066	0.208
	PE	49.7	6.3	37.3	62.0		—-	—-	—-	—-	—-	—-

**Pb [mmHg]**	NP	16.4	0.4	15.6	17.1	**MD**	0.510	0.025	–3.964	–0.484	–4.474	–3.990
	HP	15.9	2.1	11.6	20.1	**P**	0.408	0.495	0.030	0.432	0.065	0.065
	GH	16.4	1.9	12.7	20.0	**Boot. P**	0.424	0.496	0.021	0.448	0.069	0.097
	PE	20.3	2.0	16.3	24.4		—-	—-	—-	—-	—-	—-

**Pb AT [ms]**	NP	269.3	2.7	264.1	274.5	**MD**	2.355	–10.447	16.281	–12.803	13.925	26.728
	HP	266.9	15.3	236.8	297.1	**P**	0.440	0.221	0.138	0.263	0.253	0.077
	GH	279.7	13.2	253.7	305.8	**Boot. P**	0.413	0.314	0.028	0.298	0.157	0.120
	PE	253.0	14.6	224.3	281.8		—-	—-	—-	—-	—-	—-

**RM**	NP	0.41	0.01	0.39	0.43	**MD**	–0.050	0.105	–0.025	0.155	0.025	–0.130
	HP	0.46	0.06	0.35	0.57	**P**	0.191	0.017	0.325	0.018	0.372	0.029
	GE	0.31	0.05	0.21	0.40	**Boot. P**	0.291	0.071	0.359	0.088	0.412	0.094
	PE	0.44	0.05	0.33	0.54		—-	—-	—-	—-	—-	—-

**RIx**	NP	0.29	0.00	0.28	0.30	**MD**	–0.021	0.062	–0.010	0.083	0.011	–0.072
	HP	0.31	0.03	0.25	0.36	**P**	0.231	0.007	0.356	0.013	0.388	0.018
	GE	0.23	0.02	0.18	0.27	**Boot. P**	0.316	0.045	0.379	0.068	0.412	0.066
	PE	0.30	0.03	0.24	0.35		—-	—-	—-	—-	—-	—-

**Radial applanation tonometry**												
**aoSBP [mmHg]**	NP	102.80	0.53	101.76	103.84	**MD**	5.340	–3.095	–14.634	–8.435	–19.974	–11.539
	HP	97.46	3.14	91.28	103.64	**p**	0.047	0.192	<0.001	0.038	<0.001	0.009
	GE	105.90	3.51	98.99	112.80	**Boot. P**	0.025	0.217	<0.001	0.035	<0.001	0.009
	PE	117.44	3.62	110.32	124.54		—-	—-	—-	—-	—-	—-

**Pf [mmHg]**	NP	29.90	0.45	29.02	30.78	**MD**	1.494	–3.040	–5.484	–4.534	–6.978	–2.444
	HP	28.41	2.64	23.23	33.59	**p**	0.288	0.154	0.038	0.127	0.042	0.276
	GH	32.94	2.94	27.16	38.73	**Boot. P**	0.274	0.055	0.009	0.084	0.021	0.205
	PE	35.39	3.03	29.43	41.35		—-	—-	—-	—-	—-	—-

**Pb [mmHg]**	NP	13.80	0.18	13.43	14.16	**MD**	–0.192	1.541	–0.749	1.733	–0.557	–2.290
	HP	13.99	1.09	11.85	16.13	**p**	0.431	0.106	0.278	0.146	0.369	0.089
	GH	12.25	1.22	9.86	14.65	**Boot. P**	0.437	0.034	0.307	0.113	0.375	0.069
	PE	14.54	1.25	12.08	17.01		—-	—-	—-	—-	—-	—-

**Pb AT [ms]**	NP	251.91	1.35	249.24	254.57	**MD**	9.461	8.472	5.468	–0.989	–3.993	–3.004
	HP	242.44	7.97	226.77	258.12	**P**	0.121	0.174	0.278	0.467	0.372	0.404
	GH	243.43	8.90	225.94	260.93	**Boot. P**	0.116	0.081	0.234	0.462	0.368	0.384
	PE	246.44	9.17	228.41	264.46		—-	—-	—-	—-	—-	—-

**RM**	NP	0.481	0.006	0.469	0.492	**MD**	–0.034	0.113	0.080	0.147	0.115	–0.032
	HP	0.515	0.035	0.447	0.583	**p**	0.166	0.002	0.024	0.003	0.015	0.274
	GE	0.368	0.039	0.292	0.444	**Boot. P**	0.254	0.001	0.023	0.005	0.027	0.235
	PE	0.400	0.040	0.322	0.479		—-	—-	—-	—-	—-	—-

**RIx**	NP	0.321	0.003	0.315	0.326	**MD**	–0.015	0.050	0.033	0.065	0.048	–0.017
	HP	0.335	0.015	0.305	0.366	**p**	0.170	0.002	0.035	0.003	0.022	0.243
	GE	0.271	0.017	0.237	0.305	**Boot. P**	0.244	<0.001	0.026	0.001	0.033	0.200
	PE	0.288	0.018	0.253	0.323		—-	—-	—-	—-	—-	—-

NP, non-pregnant women; HP, healthy pregnant women; PAH, pregnancy-associated hypertension; GH, gestational hypertension; PE, preeclampsia; MV, mean value; SE, standard error; LL and UL, 95% confidence interval lower limit and upper limit; Boot, bootstrapping; *p*, *p*-value; aoSBP, central SBP; Pf, central forward pulse pressure Height; Pb, central backward pulse pressure height; PbAT, central backward arrival time; RM, reflection magnitude; RIx, reflection index; MD, mean difference.

### Wave separate analysis-derived indexes of non-pregnant, healthy pregnancy, and pregnancy-associated hypertension

#### Forward pressure

While women with HP showed a lower Pf compared to NP, women with PAH showed higher Pf in comparison to both HP and NP (regardless of the measurement approach, i.e., CT or RT). These differences were particularly pronounced when using CT recordings (Table 3).

When separately assessing both hypertensive states (GH and PE), Pf values tended to be higher than those measured in women with HP. Notably, there were no statistical differences in Pf between GH and PE even when using different measurement modalities (Table 3).

#### Backward pressure

There was no single case in which PAH was associated with significant differences in Pb with respect to NP or HP. There were also no differences in PbAT, regardless of the recording technique (CT or RT).

However, according to the analysis of both hypertensive states, PE showed a tendency to present with higher Pb values than NP, HP and GH, as well as a trend of having faster wave reflection arrival (i.e., lower PbAT) (Table 3).

#### Reflection magnitude and reflection index

The analysis of RM and RIx showed similarities when using CT and RT recordings. PAH presented with lower RM and RIx compared to NP and HP, although these observations were only statistically significant when using RT-derived measurements, while the threshold of significance was not reached for CT recordings. On the other hand, GH and PE showed no differences in the analysis of these parameters (Table 3).

## Discussion

### Main findings

The present work, to our knowledge, is the first one to determine and compare WSA- and PWA-derived indexes in a group of healthy NP, and HP and PAH, by using two different approaches (RT and CT). The main contributions of this study are:

First, from a methodologic standpoint, despite the existence of positive associations between the same WSA-derived indexes obtained by RT or CT, the strength of association observed in each single parameter was no more than “moderate” (Table 2). Accordingly, special care must be taken when interpreting WSA-derived indexes, as the levels of these parameters are not the same when using RT vs. CT and these techniques cannot be used interchangeably.

Second, while Pf was positively associated with the Pb level, it was negatively (very weakly, weakly, and moderately) associated with the levels of PWA-derived indexes, both when analyzing the “net” (AP, APHR75) or the “relative” (AIx, AIxHR75) contribution to wave reflections (Table 2).

Third, Pb was positively associated with AP and AIx, although the strength of association was very weak (*r* < 0.2). This provides evidence that indexes of “wave reflection” obtained by WSA and PWA are, in fact, not identifying or describing the same physiological characteristics (Table 2). Therefore, despite these parameters potentially providing complementary information about wave reflections, they hold little relationship and the information derived from these parameters should be used cautiously.

Fourth, PAH had characteristically high Pf compared to NP and HP (regardless the site of arterial tonometry recording). Subgroup analysis revealed that this finding was in fact shared by both subtypes of hypertensive states (GH and PE) (Table 3), and no differences were observed between these conditions. Consequently, the PAH state, to a good extent, is the result of a large anterograde pressure component generated by the LV itself in combination with its interaction with the arterial and microcirculatory systems, and not simply by an increase in the retrograde pressure component (wave reflections), as previously suggested. Surprisingly, PAH was not clearly associated with significant differences in Pb or PbAT compared to NP and HP, regardless of the tonometry recording site (Table 3). Thus, there is no consistent evidence that would indicate that PAH status (as a group) would represent a state characterized by higher levels of wave reflections, either by an increase in the magnitude or by an early arrival of the wave reflection from the periphery to the center. However, when analyzing PE, this group did show a trend of presenting with higher Pb than NP, HP and GH, and an earlier return of wave reflections (lower PbAT) (Table 3). It is important to note that this observation occurred without clear statistical significance in all comparisons and further studies would be needed to clarify this result. Finally, PAH status showed a trend of presenting lower RM and RIx than NP and HP, with no differences between PE and GH. When analyzing all this data together, it seems that WSA does not support the idea that PAH represents a consistent high wave reflection hemodynamic state, but to the contrary, a “high Pf state.”

In normal conditions, intermittent forward pressure waves generated by the LV collide with reflection sites located throughout the arterial tree, resulting in transmitted pressure waves (forward resultants) and pressure wave reflections (backward resultants). One of the most important reflection sources resides in the normal “stiffness gradient” of the arterial system (the farther from the LV, the stiffer the arteries get). The stiffness gradient functions as a filter of the forward pressure waves, protecting the microcirculation from high energy pressure transmission by creating wave reflections at the level where the great complaint arteries (elastic arteries) transition to the relatively smaller and stiffer muscular arteries (34). We have shown recently that when compared with NP, HP was associated with a preserved “center-to-periphery” arterial stiffness gradient (evaluated through pulse wave velocity [PWV] ratio) despite a significant drop in central aortic stiffness, quantified as the quotient between carotid-femoral PWV and carotid-radial PWV. In addition, when compared with NP and HP, PAH was associated with an “exaggerated rise” in the PWV ratio (attenuation or even reversal of the gradient), and thus, leading to a dissipation of one of the potential sources of wave reflections and microcirculation protection (7). Altogether, these observations agree with our current study. By using a methodology that is not related directly with PWV measurements (arterial stiffness assessment), our study reveals that the levels of wave reflections assessed by RM and RIx are significantly reduced in PAH, which is conceptually consistent with the aforementioned. Thus, despite the fact that women with PAH showed a higher Pf, which in turn could be associated with a higher aortic stiffness (hyperdynamic LV encountering a relatively stiff aorta during systole), this did not translate to an invariably higher Pb.

Similarly, both RM and RIx indexes also provide information about the ability of the cardiovascular system to filter excessive pressure energy transmission to certain microcirculatory beds. As mentioned, from a physiologic standpoint, the reduced RM and RIx observed in PAH would both have impaired protective effects on the distal microcirculation, potentially leading to excessive barotrauma and shear forces which would result in damage to peripheral vascular beds (e.g., placental circulation). In the setting of excessive pulsatile pressure, an increased arteriolar myogenic response could function as the last resource to protect the distal organ, but at the expense of reducing the distal peripheral perfusing blood flow. Given the fetal metabolic needs, the placenta must operate at very high flow/low vascular resistance, making it second only to the kidney regarding blood flow rates per unit of tissue mass (11). Other low-resistance vascular beds, such as renal, hepatic, and cerebral circulation can also be at risk of excessive pulsatility, since microvascular pressure is also directly coupled with aoBP fluctuations (3). Hence, the transmission of a higher pulsatile pressure into the placental and other low-resistance microcirculations might be highly likely in the setting of attenuation of RM or RIx, leading potentially to secondary placental dysfunction (e.g., intrauterine growth restriction), hepatic damage (e.g., elevated liver enzymes, hematoma), and renal damage (e.g., proteinuria), among other PE-related complications.

### Strengths and limitations

Our results should be analyzed in the context of both its strengths and limitations. To the best of our knowledge, there are no studies in the literature that have evaluated WSA-related indexes in HP and PAH. Another important strength of this study is the robustness of the methodology employed to assess WSA, utilizing two different approaches: CT and RT, which consists in a simple, non-invasive, robust, and reproducible methodology. In fact, the use of applanation tonometry has been largely validated and is regarded as the “gold standard” method for measuring waveform-derived indexes.

This study has certain limitations, however. First, since this is a cross-sectional study, it provides no data on longitudinal pregnancy-related temporal variations in the variables of interest. Second, in this work, the concept of WSA-derived indexes was presented as “static or unchanged” rather than the composite of (i) “fixed or stable” (e.g., age-dependent vascular [intrinsic] stiffness level) and (ii) “variable or adjustable” (e.g., endothelial and vascular smooth muscle ability to temporally adjust the RM or RIx level) (35). The systematization of recording conditions is necessary to evaluate WSA- and PWA-derived indexes considering the existence of modulating factors. In this work, to systematize the measurement and to minimize the impact of sources of variability, RT and CT recordings were determined at rest and under stable hemodynamic conditions, while only recordings with high operator index values (>95%) were accepted for further analysis. Third, the sample size of our group of pregnant women is relatively small. To overcome this limitation, we used bootstrapping, a statistical method that creates a new sample of observations of the variables by randomized re-sampling, with replacement based on the original observations. This method has its own advantages and disadvantages, but in this context, the biggest mistake that we can make is not generating a type I error (finding differences when in reality there are none), but, in fact, generating a type 2 error (not finding differences when in fact there are). Thus, we have taken the “conservative” approach, thereby, potentially missing significant differences that truly exist. Finally, as this study is considered exploratory and hypotheses generating (e.g., multiple correlations performed), larger sample size and/or prospective analyses will be indicated to assess and explore meaningful parameters in these patients.

## Conclusion

First, despite the existence of a positive association between one single WSA-derived index obtained by CT and RT, these associations were, in general terms, of moderate strength, so these approaches cannot be used interchangeably. Second, Pf was positively associated with Pb and negatively with PWA-derived indexes, both when analyzing the “net” (AP, APHR75) and the “relative” (AIx, AIxHR75) contribution of wave reflections. Third, Pb was positively associated with AP and AIx, although the strength of this association was very weak, which indicates that indexes of wave reflections obtained by WSA and PWA do not identify similar physiologic hemodynamic characteristics.

Fourth, PAH status was associated with higher Pf compared to HP and NP, regardless of the tonometry recording site. Both hypertensive states (GH and PE) were associated with higher Pf compared to HP, without significant differences with regards to Pb or PbAT when compared to NP or HP. However, women with PE showed a trend of presenting with higher Pb in comparison to NP, HP, and GH, with a potentially faster arrival of wave reflection components. Through our results, the use of WSA supports the idea that hypertension in women with PAH is mainly explained by a higher Pf rather than increased wave reflections.

## Data availability statement

The original contributions presented in this study are included in the article/Supplementary material, further inquiries can be directed to the corresponding author/s.

## Ethics statement

The studies involving human participants were reviewed and approved by Comité de Ética del Centro Hospitalario Pereira-Rossell. The patients/participants provided their written informed consent to participate in this study.

## Author contributions

MP, JT, DB, and YZ contributed to conception and design of the study. JT, YZ, and DB performed the cardiovascular non-invasive recordings and constructed and organized the database. YZ and DB performed the statistical analysis. MP, JT, JB, DB, and YZ wrote the first draft of the manuscript. JB, CS, and AD performed the revisions and critically discussed the complete manuscript. All authors read and approved the submitted version.

## Abbreviations

Aix: augmentation index
AIxHR75: AIx corrected for heart rate equal 75 beats/minute
aoBP: central aortic blood pressure
aoPP: aortic pulse pressure
aoSBP: aortic systolic blood pressure
AP: augmentation pressure
APHR75: AP corrected for heart rate equal 75 beats/minute
baBP: brachial artery blood pressure
baDBP: brachial artery diastolic blood pressure
baMBP: brachial artery mean blood pressure
baSBP: brachial artery systolic blood pressure
BH: body height
BMI: body mass index
BW: body weight
CRFs: cardiovascular risk factors
CT: carotid artery applanation tonometry
CVD: cardiovascular disease
DTTI: diastolic pressure or tension-time area or index
GH: gestational hypertension
GTF: general transfer function
HP: healthy pregnancy
HR: heart rate
NP: non-pregnant
PA: physical activity
PAH: pregnancy-associated hypertension
Pb: amplitude of central backward blood pressure component
Pb AT: central backward pressure arrival time to central pressure waveform
PE: preeclampsia
Pf: amplitude of central forward blood pressure component
PWA: pulse wave analysis
RIx: reflection index
RM: reflection magnitude
RT: radial artery applanation tonometry
SEVR: subendocardial viability ratio
STTI: systolic pressure or tension-time area or index
WSA: wave separation analysis.
