# Supplementary material for: Wave separation analysis-derived indexes obtained from radial and carotid tonometry in healthy pregnancy and pregnancy-associated hypertension: Comparison with pulse wave analysis-derived indexes
Source: Front Cardiovasc Med. 2022 Nov 1;9:997452. doi: 10.3389/fcvm.2022.997452 (PMC9664074; doi:10.3389/fcvm.2022.997452)
Supplement: Supplementary file 1 [file Table_1.pdf]

Table S1. PWA-derived and WSA-derived indexes obtained by applanation tonometry.

Parameter	Equation and/or description	Significance
AP	• PP-P1<br>• The contribution that the wave reflection makes to the SBP or PP.	• It is a measure of wave reflection and arterial stiffness.<br>• ↑Arterial stiffness leads to an earlier return of wave reflections during systole causing an additional rise in SBP (↑AP).<br>• ↑AP may lead to ↑LV afterload ↓DBP and impaired coronary perfusion.
AIx	• AP/PPx100<br>• The relative contribution of wave reflection pressure augmentation to the SBP or PP.	• It is a measure of wave reflection and arterial stiffness.<br>• ↑AIx may lead to ↑LV afterload ↓DBP and impaired coronary perfusion.
Pf	• Forward pressure<br>• It is the amplitude of the forward pressure component when decomposing the aoBP wave.	• Represents the integration of the forward wave arising from the LV and re-reflections of backward propagating waves at the LV-aorta interface.<br>• Related with the LV stroke volume, arterial stiffness and LV afterload.
Pb	• Backward pressure<br>• It is the amplitude of the backward pressure component when decomposing the AoBP wave.	• Represents an estimate of arterial pressure wave reflection.<br>• Related with reflection sites, arterial stiffness and vascular resistance.
RM	• Pb/Pf<br>• It is an indicator of wave reflections	• Assesses the relative contribution of Pb to the resultant pressure wave.<br>• Complex parameter of wave reflection
RIx	• Pb/(Pf+Pb)<br>• It is an indicator of wave reflections	• Assesses the relative contribution of wave reflection to the net aoBP.<br>• Complex index of wave reflection.
DTTI	The AUC (tension-time integral) of the diastolic portion of the central aortic pulse wave.	• Provides information of myocardial oxygen supply.<br>• Considers both coronary driving pressure and diastolic time, indicating potential subendocardial blood supply.
STTI	The AUC (tension-time integral) of the systolic portion of the central aortic pulse wave.	• Provides information of myocardial oxygen demand.<br>• Indicates myocardial contraction.
SEVR	• DTTI/STTI<br>• Tension-time integral ratio	Index of myocardial oxygen supply and demand.

For more information about these parameters, please refer to Figure 1. *Both AP and AIx can also be normalized to HR 75 (i.e., APHR75 and AIxHR75). Abbreviations as in text.
